# Primary Intraventricular Amelanotic Melanoma: Case Report and Literature Review

**DOI:** 10.7759/cureus.99205

**Published:** 2025-12-14

**Authors:** Carlos Mávita Corral, Flavio Hernandez-Gonzalez, Kevin S Toache, Marco A Rodriguez-Florido, Luis A Gallego Hermosillo, Alejandro Suarez-Ramirez, Pedro A Gonzalez-Zavala

**Affiliations:** 1 Neurosurgery, Hospital de Especialidades, Centro Medico Nacional SXXI, Mexico City, MEX; 2 Neuropathology, Hospital de Especialidades, Centro Médico Nacional Siglo XXI, Mexico City, MEX; 3 Neurosurgery, Hospital de Especialidades Unidad Médica de Alta Especialidad No. 71, del Instituto Mexicano del Seguro Social, Torreón, MEX; 4 Neurosurgery, Hospital de Especialidades, Centro Médico Nacional Siglo XXI, Mexico City, MEX

**Keywords:** amelanotic melanoma, atrial tumor, immunohistochemistry, intraventricular tumor, primary cns melanoma

## Abstract

Primary amelanotic melanoma of the central nervous system (CNS) represents an exceedingly rare neoplasm within the category of primary CNS melanocytic tumors. The absence of melanin pigmentation often complicates its identification, necessitating histopathological and immunohistochemical evaluation for accurate diagnosis.

We describe the case of a 70-year-old man who presented with Parkinsonian features and was found to have a well-circumscribed intraventricular mass in the left atrium of the lateral ventricle (38×45×29 mm), hypointense on T1-weighted MRI with heterogeneous enhancement. Surgical resection was performed via a trans-intraparietal sulcus approach. Histopathology confirmed amelanotic melanoma, supported by immunopositivity for S-100, Melan-A, and HMB-45. The patient received adjuvant whole-brain radiotherapy followed by immunotherapy with nivolumab and ipilimumab. Although he initially improved, tumor recurrence occurred within five months, and treatment-related hepatotoxicity ultimately resulted in fatal hepatic failure one year after surgery.

This case highlights the diagnostic challenges and aggressive behavior of primary intraventricular amelanotic melanoma. Even with gross total resection and multimodal therapy, the prognosis remains poor. Early recognition and multidisciplinary management are essential in addressing this rare entity.

## Introduction

Primary melanocytic lesions of the central nervous system (CNS) are extremely uncommon, comprising 0.06-0.1% of intracranial tumors and arising from leptomeningeal melanocytes. They range from diffuse entities, such as melanocytosis, to well-circumscribed lesions including melanocytoma, intermediate-grade melanocytic neoplasms, and melanoma [1-5]. Primary CNS melanoma represents a minority within this spectrum, accounting for 1% of all melanomas and 0.07% of brain tumors, with an estimated incidence of 0.005 per 100,000 individuals [6-7]. These tumors may occur anywhere along the neuroaxis, most often in the spinal canal, Meckel’s cave, or posterior fossa [3], and melanocytomas and melanomas collectively represent 0.06-0.1% of meningeal tumors, typically presenting in the fourth or fifth decade of life [6].

Amelanotic melanomas lack significant melanin pigmentation and constitute 0.4-27.5% of cutaneous melanomas, although truly amelanotic variants account for <2% [8,9]. Primary amelanotic melanomas of the CNS are exceptionally rare, with only 16 cases reported [10-23].

Given their rarity and the diagnostic difficulty posed by nonspecific imaging features and variable clinical manifestations, documenting additional cases remains essential. Here, we report a case of primary intraventricular amelanotic melanoma in a 70-year-old man and review current evidence on presentation, anatomical location, histopathology, treatment, and outcomes.

## Case presentation

A 70-year-old man with a remote history of resection of a pigmented skin lesion on the left lower back 38 years earlier, without available histopathological documentation, presented for evaluation. Over the previous 15 years, he had developed progressive neuropsychiatric symptoms, including mood instability, irritability, anxiety, self-harming behavior, and memory decline. Two years before admission, he began to exhibit a magnetic gait, followed 18 months later by a stress-exacerbated left-hand tremor. He was recently diagnosed with Parkinson’s disease and initiated on levodopa-carbidopa.

**Figure 1:**
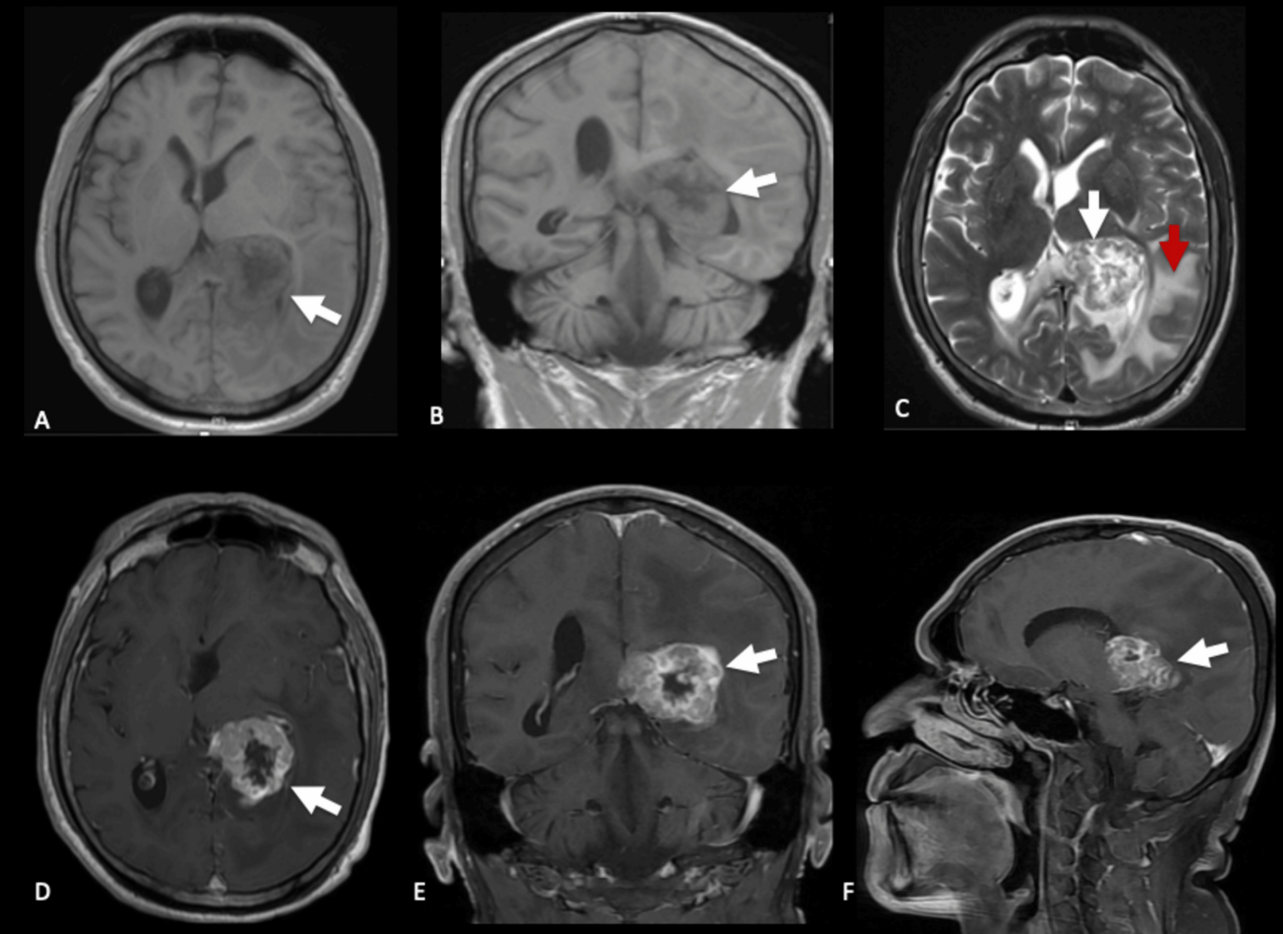
Preoperative brain MRI (A, B) Axial and coronal non-contrast T1-weighted images showing a hypointense, heterogeneous intraventricular mass in the left lateral ventricle atrium (white arrows). (C) Axial T2-weighted image demonstrating mixed signal intensity with hypointense and hyperintense components (white arrow) and surrounding vasogenic edema (red arrow). (D–F) Axial, coronal, and sagittal post-contrast T1-weighted images depicting heterogeneous peripheral enhancement (white arrows), consistent with necrotic or hemorrhagic tumor areas.

Brain MRI revealed an intraventricular, well-circumscribed lesion with irregular margins, hypointense and heterogeneous on T1, located in the left lateral ventricle atrium, measuring 38×45×29 mm. The lesion showed predominantly peripheral heterogeneous contrast enhancement. On T2-weighted images, the center was hyperintense, and the margins were hypointense, with perilesional edema in the ipsilateral temporal, parietal, and occipital white matter, along with a 15 mm midline shift and leftward dilation of the temporal horns (Figure 1).

The patient was admitted and started on steroid therapy with dexamethasone 8 mg every 8hr. On arrival, the patient had hypomimia and was alert, attentive, and oriented with decreased voice tone and memory impairment; however, the rest of the mental functions were intact. The cranial nerve examination was unremarkable. Motor evaluation, the patient demonstrated increased muscle tone in the left upper limb and cervical region, mild right hemiparesis was observed, with proximal muscle strength assessed at 4+/5 and distal strength at 4-/5 according to the Medical Research Council (MRC) muscle strength scale. Additionally, a resting tremor was noted in the left hand.

**Figure 2:**
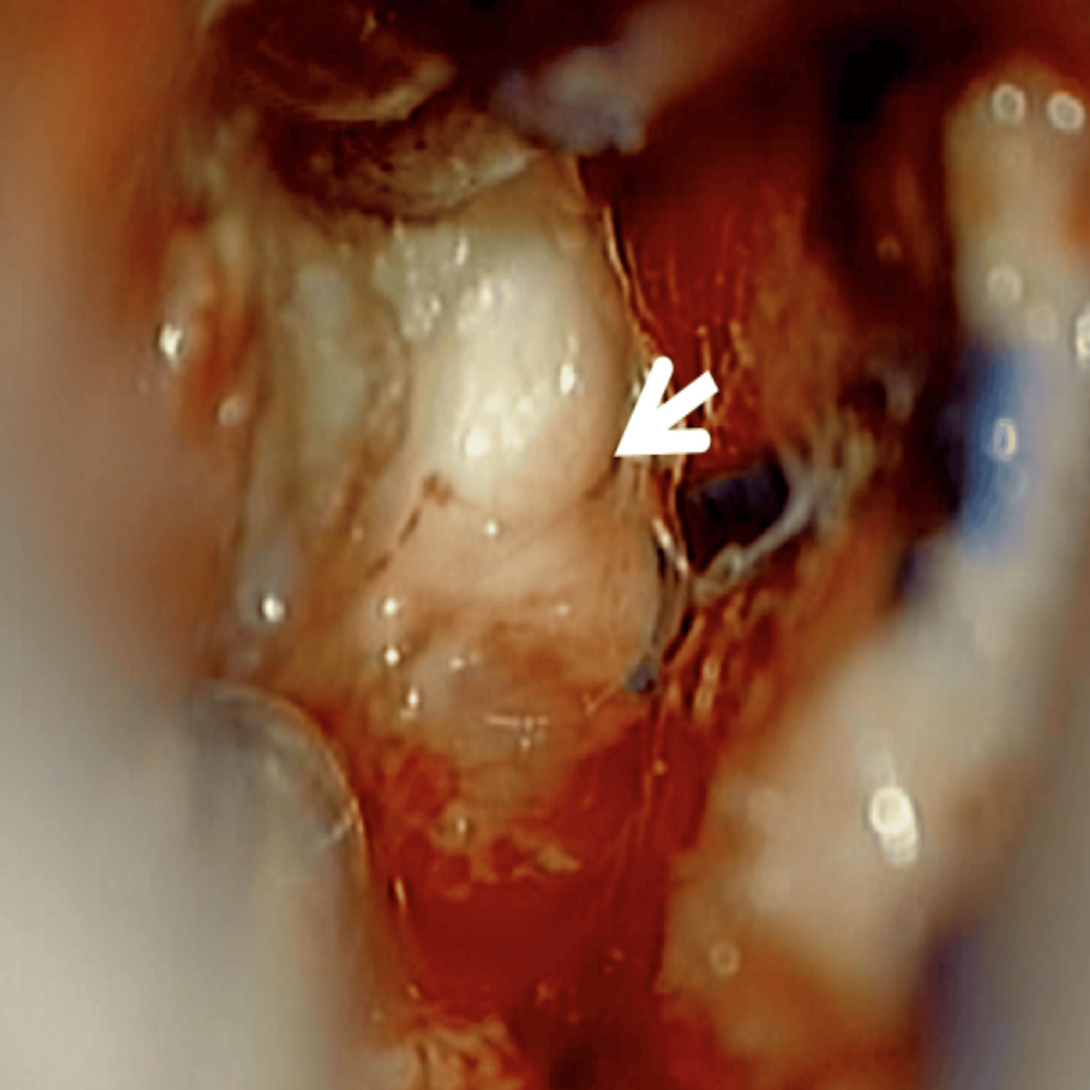
Intraoperative macroscopic appearance of the lesion. Intraoperative view through a trans-intraparietal sulcus approach showing a non-pigmented, whitish, fibrous, and partially aspiratable intraventricular mass (arrow). The lesion demonstrated a firm consistency with poorly defined margins and absence of melanin pigmentation, consistent with an amelanotic melanoma.

The patient consented to a surgical intervention initially suspected to address a ventricular atrial meningioma. A horseshoe-shaped incision and centered craniotomy were executed, followed by an intraparietal transsulcal approach to access the ventricular atrium. The surgical excision of a solid tumor, which was firmly adhered to the walls of the ventricular trigone, was successfully completed. Macroscopically, the lesion was non-pigmented (Figure 2).

**Figure 3:**
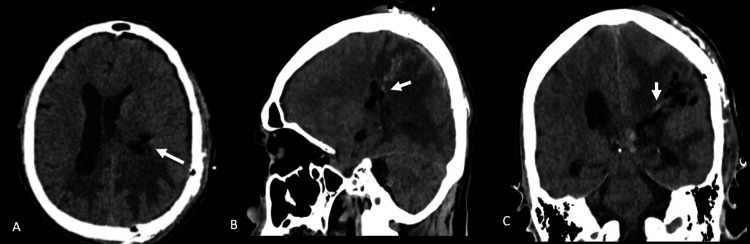
Immediate postoperative CT scan (A) Axial, (B) sagittal, and (C) coronal CT images showing small intraventricular air and minimal blood products within the left lateral ventricle atrium (white arrows), without parenchymal hemorrhage or ventricular dilation. No postoperative hydrocephalus is observed.

The procedure was conducted without significant intraoperative hemorrhage, and the patient did not require postoperative external ventricular drainage. An immediate postoperative CT scan confirmed complete resection and absence of hemorrhage or hydrocephalus (Figure 3).

The patient recovered full muscle strength in the right hemibody within the following week but continued to show symptoms of Parkinsonism.

**Figure 4:**
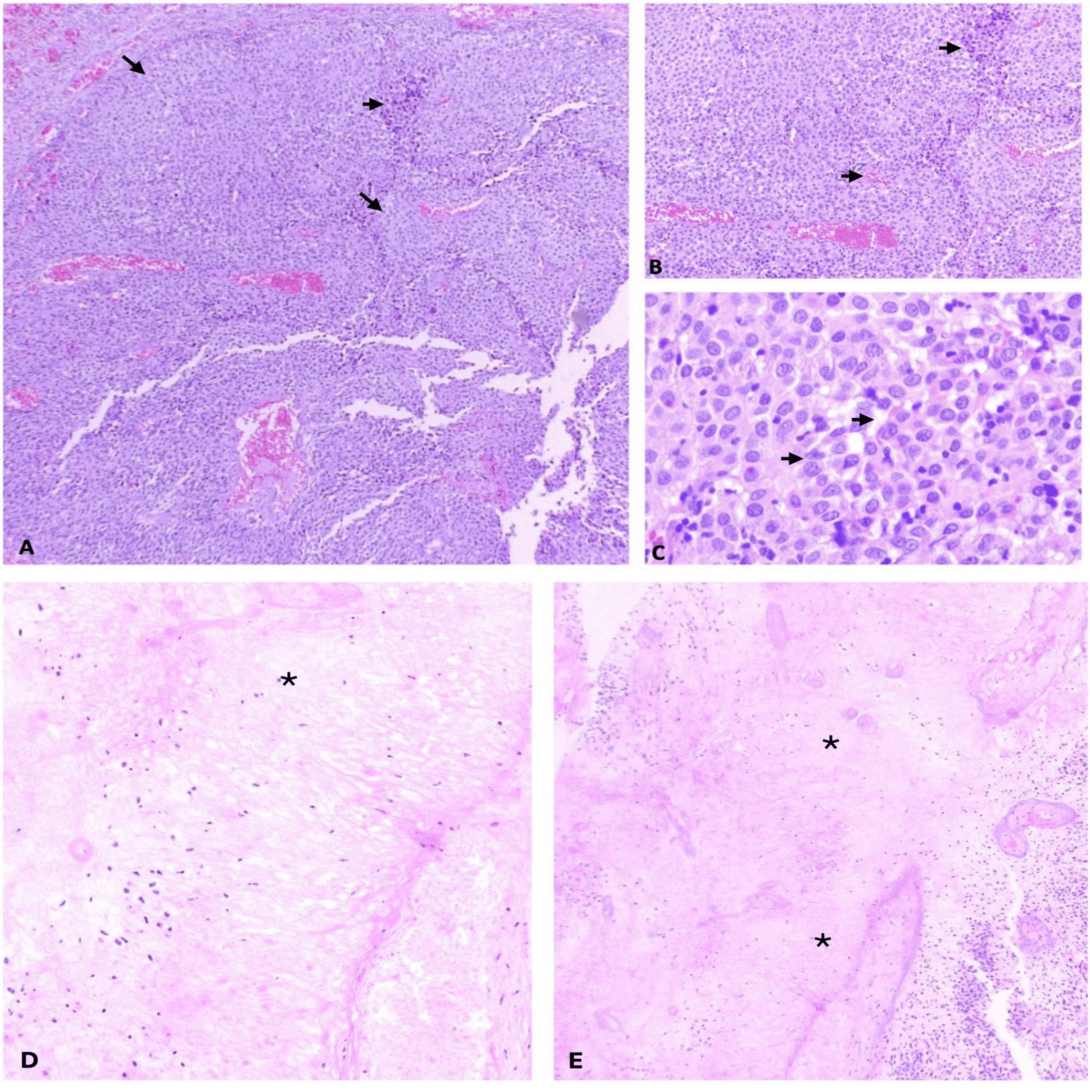
Histopathological features of a primary intraventricular amelanotic melanoma. (A) Low-power hematoxylin and eosin (H&E)–stained section shows a solid, highly cellular neoplasm with a sheet-like growth pattern and prominent vascularization (arrows). (B) Intermediate magnification demonstrates nests of uniform ovoid tumor cells arranged in perivascular distribution with preserved cohesion and scant intervening stroma (arrows). (C) High-power view reveals polygonal cells with moderate eosinophilic cytoplasm, finely dispersed chromatin, inconspicuous nucleoli, and mild nuclear pleomorphism (arrowheads). No melanin pigment is identified, consistent with an amelanotic phenotype. (D, E) Low-power panoramic views highlight extensive necrotic areas (asterisks) and adjacent viable tumor tissue.

Intraoperative histopathological evaluation revealed a high-grade, undifferentiated neoplasm. Macroscopically, the specimen consisted of multiple irregular, smooth-surfing tissue fragments with light brown coloration and papillary architecture. Hematoxylin and eosin-stained sections revealed solid neoplastic proliferation arranged in sheets. The tumor cells were ovoid, with moderate eosinophilic cytoplasm and ovoid nuclei exhibiting fine chromatin and mild nuclear pleomorphism (Figure 4).

**Figure 5:**
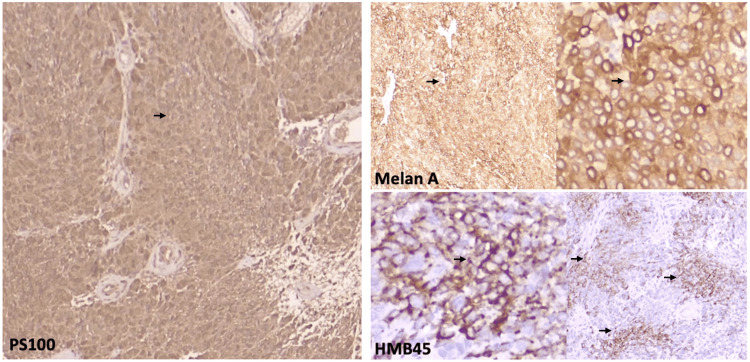
Immunohistochemical profile of the primary intraventricular amelanotic melanoma. Immunohistochemistry shows diffuse S-100 positivity (left, arrow), along with strong Melan-A expression (top right, arrows) and focal HMB-45 staining (bottom right, arrows). This melanocytic marker profile, in the absence of melanin pigment on hematoxylin-eosin sections, supports the diagnosis of amelanotic melanoma.

Immunohistochemical analysis showed diffuse positivity for S-100 protein, Melan-A, and HMB-45 in a patchy distribution, vimentin, and a Ki-67 proliferation index of 7% (Figure 5).

The tumor cells were negative for progesterone receptor, epithelial membrane antigen (EMA), somatostatin receptor 2A (SSTR2A), pancytokeratin AE1/AE3, CK5/6, CK7, CK20, p63, placental alkaline phosphatase (PAGF), thyroid transcription factor-1 (TTF-1), and synaptophysin (Table 1).

**Table 1:** Immunohistochemical markers and their diagnostic significance. The diagnosis of amelanotic melanoma was supported by the combined immunohistochemical positivity for S-100, Melan-A, and HMB-45, confirming melanocytic origin despite the absence of visible pigmentation. *Abbreviations:* CK: Cytokeratin; EMA: Epithelial Membrane Antigen; HMB-45: Human Melanin Black-45; Ki-67: Kiel-67 proliferation index; MART-1: Melanoma Antigen Recognized by T cells 1; PAGF: Placental Alkaline Phosphatase; S-100: S-100 protein; SSTR2A: Somatostatin Receptor 2A; TTF-1: Thyroid Transcription Factor-1. (Non-abbreviated markers: Melan-A, p63, Synaptophysin, Vimentin, Progesterone receptor, Pancytokeratin AE1/AE3, CK5/6, CK7, CK20.)

Marker	Result	Diagnostic Significance
S-100	Positive	Highly sensitive marker for melanocytic differentiation; supports the diagnosis of melanoma.
Melan-A (MART-1)	Positive	Specific melanocytic marker; indicates melanocytic lineage.
HMB-45	Positive (patchy)	Specific for immature melanosomes; supports melanoma, including metastatic or amelanotic variants.
Vimentin	Positive	Mesenchymal marker commonly expressed in melanomas; supportive but non-specific.
Ki-67 (7%)	Positive	Indicates proliferative index; low–intermediate proliferation consistent with some metastatic melanomas.
Progesterone receptor	Negative	Helps exclude meningioma.
EMA	Negative	Further argues against meningioma or epithelial tumors.
SSTR2A	Negative	Helps rule out meningioma, which typically shows strong positivity.
Pancytokeratin AE1/AE3	Negative	Excludes carcinoma or epithelial metastases.
CK5/6, CK7, CK20	Negative	Further excludes carcinomas of various origins.
p63	Negative	Helps rule out squamous cell carcinoma.
PAGF (placental alkaline phosphatase)	Negative	Argues against germ cell tumors.
TTF-1	Negative	Helps exclude lung or thyroid metastatic carcinoma.
Synaptophysin	Negative	Excludes neuroendocrine tumors.

Clinical, imaging, and histopathological findings supported the diagnosis of primary amelanotic melanoma. A PET-CT scan was initially planned to evaluate for systemic involvement and to exclude an extracranial melanoma source; however, it could not be performed due to technical failure of the equipment at that time. Instead, a contrast-enhanced thoracoabdominal-pelvic CT scan was obtained, which revealed no suspicious lesions or lymphadenopathy suggestive of another primary melanoma or metastatic disease.

**Figure 6:**
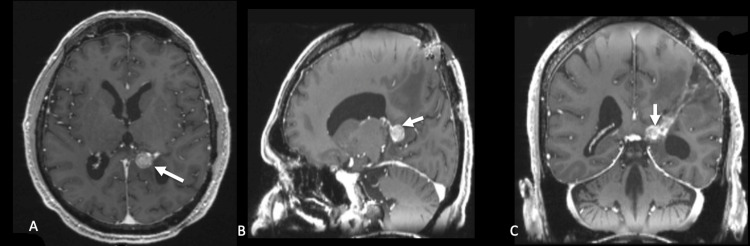
Follow-up MRI (A) Axial, (B) sagittal, and (C) coronal post-contrast T1-weighted images show a newly developed enhancing nodular lesion in the left lateral ventricle atrium (white arrows), consistent with local tumor recurrence five months after surgery.

The patient received adjuvant whole-brain radiotherapy (30 Gy in 10 fractions) followed by combined immunotherapy with nivolumab (3 mg/kg every 15 days) and ipilimumab (1 mg/kg every 21 days for four cycles). Five months post-surgery, follow-up MRI revealed local tumor recurrence in the left ventricular atrium, characterized by new nodular enhancement (Figure 6), indicative of aggressive progression. Although treatment was ongoing, the disease continued to advance, and the patient's clinical condition steadily deteriorated. Six months after starting immunotherapy, he experienced immune-related liver damage, indicated by elevated liver function tests: total bilirubin at 6.42 mg/dL (reference: 0.1-1.2 mg/dL), AST at 979 U/L (reference: 5-30 U/L), and ALT at 1273 U/L (reference: 4-36 U/L), which necessitated the cessation of immunotherapy. Ultimately, the patient passed away from the illness one year following surgery.

## Discussion

Central nervous system (CNS) melanocytic tumors are rare neoplasms that may correspond to metastases or, less commonly, to primary lesions [1,24,25]. The latter includes both benign and malignant tumors, which can be either localized or diffuse [1,16,25].

Melanocytic neoplasms originate from melanocytes in the neural crest during early embryogenesis. Melanoblasts migrate dorsolaterally to the skin during the first trimester of gestation. A smaller number of cells travel to mucosal surfaces, such as the aerodigestive and urogenital tracts, inner ear, uvea, and leptomeninges. Therefore, these neoplasms can arise in cutaneous or extracutaneous locations, with the latter being less common [1].

Primary melanomas of the CNS account for less than 1% of all melanomas. Although they can occur anywhere in the nervous system, they are more commonly found in the posterior cranial fossa, spinal cord, and Meckel's cave [3,6,7]. They typically appear between the ages of 15 and 71 years, with a peak incidence in the fifth decade of life [6]. Diagnosis is not straightforward, as it requires the exclusion of systemic melanoma [26].

An unusual variety of melanocytic lesions that seems to defy the nature of this tumor by definition is the amelanotic variant. It lacks melanin pigment or, depending on the definition, may present it in less than 5% of the cells [15]. Reports of amelanotic specimens are rare among melanomas [15]. The clinical features of amelanotic melanoma are similar to those of melanocytic lesions, which are closely related to the tumor's anatomical location and compression of nearby nerves. They commonly present with intracranial hypertension, hydrocephalus, focal neurological deficits, subarachnoid hemorrhage, and seizures [15,27,28].

Magnetic resonance imaging (MRI) is the preferred imaging method for melanoma in the central nervous system; however, its performance is influenced by the amount of melanin and the presence of hemorrhage. Isiklar et al. classified MRI performance into four groups: a) The melanotic group, which shows hyperintensity on T1 and hypointensity on T2; b) Amelanotic group, with iso-/hypointensity on T1 and iso-/hyperintensity on T2. c) Mixed group: does not meet either of the two criteria. d) Hemorrhagic group with features of intra/peritumoral hemorrhage [15,29,30].

In addition to CT and MRI, PET/CT is used as an imaging method for central nervous system melanoma. PET/CT can help MRI clarify the primary lesion and identify metastases [27]. Histologically, primary melanoma cells can present a polygonal or spindle-shaped morphology with marked heterogeneity and abundant mitotic activity. Some tumors may exhibit signs of hemorrhage and necrosis. According to case report findings, the Ki67 proliferation index in primary CNS melanoma ranges from 1% to 30%. HMB-45, Melan-A, and S-100 are specific markers of melanoma. HMB-45 and Melan-A showed limited sensitivity (69%-93% and 75%-92%, respectively), whereas S-100 showed high sensitivity (97%-100%) but low specificity (75%-87%) [27].

In primary CNS melanoma cells, positive expression of HMB45, S-100, and Melan A is observed, while meningeal epithelial membrane antigen, cytokeratin, neuron-specific enolase, and glial fibrillary acidic protein are expressed negatively. This expression pattern is valuable for distinguishing primary CNS melanoma from other tumors. GNAQ and GNA11 mutations are prevalent in primary CNS melanoma but differ from cutaneous melanoma (CM), in which BRAF, NRAS, KIT, and NF-1 mutations are more common [1,8,24,27].

The study of metastatic leptomeningeal melanomas has shown a highly immunosuppressive microenvironment enriched with T-cell populations, with a low percentage of CD4 and NK T-cells but a high percentage of CD8 T-cells. In cases of brain metastatic melanoma, a higher number of activated CD4 cells has been observed [27].

Regarding treatment, resection, radiotherapy, chemotherapy, and biotherapy have shown variable responses as single or combined agents. However, owing to the infrequent and under-researched nature of primary CNS melanomas, an optimal standard treatment has not been defined [3,26].

In a population-based study, Puyana and collaborators analyzed 54 cases of primary malignant CNS melanoma between 1973 and 2015, finding that total macroscopic resection plus radiotherapy and both plus chemotherapy were significantly associated with higher survival rates compared to biopsy [26].

Total macroscopic resection is considered by most authors to be the cornerstone of treatment, as it has significant effects on survival, being especially useful in solitary lesions or large and/or symptomatic metastases with edema [31]. Radiotherapy and chemotherapy have been recommended, whereas partial resection has shown less clear effects on survival [26]. Radiotherapy has shown greater potential as a primary therapy for disseminated CNS melanomas or as an adjuvant post-surgery to prevent progression or recurrence [26]. With single-fraction stereotactic radiosurgery (18 Gy) plus immunotherapy, 12-month control rates for a 7.5 mm lesion were 87.8% compared to 79.8% for radiosurgery alone [32]. Systemic chemotherapy is a treatment for advanced CNS melanoma. The median progression-free survival in patients receiving systemic chemotherapy is approximately five months, and the overall survival is approximately 12.5 months. Currently, chemotherapy regimens for primary CNS melanoma have not been standardized yet. Temozolomide, dacarbazine, platinum-based drugs, and intrathecal methotrexate are commonly used, although most of these chemotherapeutic agents do not cross the blood-brain barrier, with the exception of temozolomide, which has provided survival benefits for some patients [3,27]. Advances in the molecular genetics of tumors have marked the beginning of the era of targeted therapies, which have shown significant clinical benefits in patients with advanced melanoma [27].

Immunotherapy has proven to be promising in improving survival outcomes in advanced melanoma, particularly in cutaneous melanoma. Guo and collaborators collected data on five cases of primary CNS melanoma treated with immunotherapy, who achieved a median overall survival of 58 months (5 to 63 months). These findings indicate the partial efficacy of immunotherapy in treating CNS melanoma and suggest the possibility of greater efficacy when combined with other treatment strategies [27]. Currently, ipilimumab plus nivolumab is the first-line treatment for patients with asymptomatic brain metastases from melanoma. [33]. With a 10-year follow-up, the median overall survival was 71.9 months with this combination treatment. Among patients alive and progression-free at 3 years, the 10-year melanoma-specific survival rate was 96% [34].

Literature review 

To better characterize the clinical and pathological features of this rare variant, a literature review was performed, identifying 16 previously reported cases of primary CNS amelanotic melanoma (Table 2). The patients had an average age of 47.3 years. The symptoms varied based on where the tumor was located, with cognitive issues being among the most commonly noted. None of the cases mentioned any preoperative suspicion of the diagnosis. The lesions were reported in various locations, including the brainstem, hemispheric parenchyma, meninges, spine, cerebellum, sella turcica, parasellar region, cranial cavity, and there was one case of a suspected intraventricular lesion.

**Table 2:** Reported cases of primary central nervous system amelanotic melanoma in the literature. Abbreviations: BRAF V600E: B-Raf proto-oncogene, serine/threonine kinase (V600E mutation); GNA11: guanine nucleotide-binding protein subunit alpha-1; HMB-45: human melanoma black-45; MART-1: melanoma antigen recognized by T-cells 1; S100: S100 calcium-binding protein; SMA: smooth muscle actin.

Reference	Gender	Age	Location	Clinical symptoms	Histopathology	Treatment	Evolution
Shuknecht et al 1990 [19]	Male	53 y	Left parietooccipital meningeal	Seizures and headache, acute bleeding	Polymorphic Vimentin +, HMB45 +++, S100 +++	Craniotomy and adjuvant radiotherapy.	5 month recidive, 18 month survival. Reintervention and intrathecal chemotherapy
Shuknecht et al 1990 [19]	Male	23 y	Right temporal meningeal	Seizures and headache	Not Specified	Temporal pole lobectomy, radiotherapy and intrathecal chemotherapy	4 month survival
Seki et al 1991 [21]	Male	38 y	Left parietal intraventricular	Headache, memory loss, right hemiparesis, right hemianopsia	Atypical melanosomes in electronic microscopy	Craniotomy and resection	1 month survival
Vanzieleghem et al 1999 [23]	Female	2 y	Right mesencephalic	Hydrocephalus, right ptosis, left eye palsy	Positivity for HMB45, S100	Ventriculoperitoneal shunt, Biopsy	1 month survival
Li et al 2004 [14]	NS	NS	Not Specified	Not Specified	Not Specified	Not Specified	Not Specified
Jacob et al 2006 [13]	Female	63 y	Sella and cavernous sinus	Diplopia and bilateral ptosis	Positive for S100, Melan-A and HMB45	Transsphenoidal hypophysectomy and debulking	Progression to blindness and death
Schulz et al 2012 [20]	Male	72 y	Right cerebellar hemispheric, left temporal, thoracic intradural	Headache, nausea, vomit, and gait ataxia	Positivity HMB45, S100 Negativity for Melan A	Craniotomy for cerebellar lesion, resection of spinal lesions	Not Specified
Said et al 2014 [18]	Male	60 y	Left frontal	Numbness and dizziness	BRAF V600 mutation	Biopsy and chemotherapy	Not Specified
Ma et al 2015 [15]	Male	64 y	Right frontal	Headache, mass in frontal region	Intermediate grade Positivity for HMB45, MelanA, S100, Vimentin, CD99, SMA, CD34	Right frontal craniotomy	5 month tumor free
Szathmari et al 2016 [22]	Female	5 y	Cystic enlargement of basal cisterns, leptomeningeal, left parietal	Headache, vomiting	Positivity for HMB45, MelanA	Occipitoaxial decompression, endoscopic cystostomy and biopsy, ventriculoperitoneal shunt, chemotherapy	11 month survival
Aslan et al 2018 [11]	Female	44 y	Intracranial and spinal leptomeninges	Nausea, vomiting, weight loss, cognitive deterioration, paraparesis, incontinence	Positivity for S100, HMB45, MelanA	Biopsy, chemotherapy	4 month survival
Mayer et al 2018 [16]	Male	71 y	Cerebellum and spinal metastases (intramedullary T11, epidural L2)	Difficulty for concentration and hand coordination, bilateral dysmetria and unsteady gait	Pleomorphism Positivity for vimentin and HMB45 Negativity for MelanA Ki67 40%	Midline suboccipital approach, radiation	Hydrocephalus, treated with ventriculoperitoneal shunt
Zhang et al 2019 [24]	Female	26 y	Posterior fossa meninges, leptomeningeal dissemination	Headache and nausea	Positivity for HMB45, S100, MART1 Ki67 30% BRAF V600E	Right suboccipital craniotomy	10 month survival
Chaharyn et al 2022 [12]	Female	64 y	Cervicomedullary junction	Gait difficulties, dysesthesias	Not Specified	Variant of GNA11 gene	Not Specified
Andrés Sanz et al 2022 [10]	Female	63 y	Right frontal	Headache, unstable gait, behavioral changes, right homonymous hemianopia, paraparesis, left facial paralysis	Positivity for HMB45, Vimentin, p53, CD99, S100 Ki 60% Negativity for BRAF V600E	Right frontal craniotomy	Status epilepticus, 3 month survival
Polster et al 2023 [17]	Male	62 y	Right frontal	Confusion and left hemiparesis	BRAF V600E mutation	Transorbital transcaruncular	No visual symptoms and good cosmetic outcome at 3 months

Pathological findings showed positivity for HMB-45 in 50% of the cases, S-100 positivity in 37.5% of the cases, and MelanA positivity in 31.25% of the cases; we associated these low percentages with the fact that immunochemistry was not performed or mentioned in the case reports. The surgical approach depends on the location and clinical features of the case. The prognosis is generally unfavorable, with an average survival of 6.5 months.

In the present case, an amelanotic melanoma was found in an unusual intraventricular location, initially suspected to be an atrial meningioma. Optimal treatment was achieved with gross total resection and adjuvant radio- and chemotherapy. The patient had a one-year survival but died due to chemotherapy-related complications. Although not widely studied due to the rarity of the amelanotic variant, available evidence suggests that prognosis and survival are worse compared to pigmented melanomas.

## Conclusions

Primary intraventricular amelanotic melanoma represents an exceedingly rare and complex central nervous system tumor, necessitating a comprehensive exclusion of extracranial primary sites and relying on immunohistochemical analysis for definitive diagnosis. Despite advancements in therapeutic strategies, the prognosis remains unfavorable due to high recurrence rates and treatment-associated toxicity. This case highlights the tumor's aggressive characteristics and underscores the necessity for thorough evaluation and multidisciplinary management.
